# CIRCULATING MIRNA PROFILING FOR THE DETECTION OF SARCOPENIA COMPONENTS IN OLDER ADULTS

**DOI:** 10.1093/geroni/igae098.3642

**Published:** 2024-12-31

**Authors:** Hyung Eun Shin, Jae Young Jang, Daehyun Lee, Heeeun Jung, Hyunjin Cho, Nahyun Lim, Chang Won Won, Miji Kim

**Affiliations:** Kyung Hee University, Seoul, Republic of Korea; Kyung Hee University, Seoul, Republic of Korea; Kyung Hee University, Seoul, Republic of Korea; Kyung Hee University, Seoul, Republic of Korea; Kyung Hee University, Seoul, Republic of Korea; Kyung Hee University, Seoul, Republic of Korea; Kyung Hee University, Seoul, Republic of Korea; Kyung Hee University, Seoul, Republic of Korea

## Abstract

MicroRNAs (miRNAs), short, non-coding RNAs that regulate gene expression at translation, are recently emerging as potential biomarkers for sarcopenia. The miRNAs have been extensively studied in etiology of sarcopenia; however, the role of miRNAs on sarcopenia components has not yet been explored. We aimed to examine the differentially expressed miRNAs in plasma of older adults in relation to muscle mass, strength, and performance status. A total of 96 older adults (women: 50.0%; mean age: 76.6 ± 3.6 years) were randomly selected and matched by age and sex from the Korean Frailty and Aging Cohort Study (KFACS). Participants were categorized into four groups based on criteria of Asian Working Group for Sarcopenia 2019: “Normal” (n = 25), “Low muscle mass (LMM) only” (n = 23), “Low muscle strength (LMS) only” (n = 25), “Low physical performance (LPP) only” (n = 23). MiRNA profiles, derived from miRNA sequencing, were established via screening for differentially expressed miRNAs in four groups, and candidate differentially expressed miRNAs were validated using RT-qPCR. The miRNA profiling and validation analysis showed that three differentially expressed miRNAs, including miR-144-3p, miR-122-3p, and miR-210-3p, were significantly increased in plasma of “LMS only” group, compared to “LMM only” and “LPP only” groups (p < 0.05). The Kyoto Encyclopedia of Genes and Genomes revealed that three miRNAs were mainly related to neurotrophin signaling pathway (p < 0.05). These results suggest that increased levels of miR-144-3p, miR-142-3p, and miR-122-3p could serve as candidate plasma biomarkers for the decline of muscle strength in older adults.

